# Identification of two biological subgroups of complex regional pain syndrome type 1 by transcriptomic profiling of skin and blood in women

**DOI:** 10.1186/s10020-025-01148-y

**Published:** 2025-03-12

**Authors:** Melina Pérez Vertti Valdés, Astrid Jüngel, Pamela Bitterli, Jan Devan, Hubert Rehrauer, Lennart Opitz, Laura Sirucek, Petra Schweinhardt, Sabrina Catanzaro, Oliver Distler, Florian Brunner, Stefan Dudli

**Affiliations:** 1 Center of Experimental Rheumatology, Balgrist Campus, University Hospital Zurich, University of Zurich, Lengghalde 8, 8008 Zurich, Switzerland; 2 Functional Genomics Center Zurich, ETH Zurich and University of Zurich, 8057 Zurich, Switzerland; 3 Department of Chiropractic Medicine, Integrative Spinal Research Group, Balgrist University Hospital, University of Zurich, Zurich, Switzerland; 4 Unit of Clinical and Applied Research, Balgrist University Hospital, University of Zurich, Switzerland, Zurich, Switzerland; 5 Department of Physical Medicine and Rheumatology, Balgrist University Hospital, University of Zurich, Zurich, Switzerland; 6 Center for Neuroplasticity and Pain (CNAP), Aalborg University, Aalborg, Denmark

**Keywords:** Complex regional pain syndrome (CRPS), RNA-seq, Peripheral blood mononuclear cells (PBMCs), Cytokine quantification, Chronic pain

## Abstract

**Background:**

Patients with Complex Regional Pain Syndrome (CRPS) present prolonged, debilitating pain and functional impairment. Treatments are not disease-modifying due to the poorly understood underlying pathomechanisms. This study aimed to identify the molecular signatures of potential CRPS type 1 subgroups.

**Methods:**

Twelve women with CRPS type 1 were included. Demographics and pain questionnaires were recorded. Skin biopsies of the affected and non-affected limbs (n = 6 + 6) and peripheral blood (n = 11) were collected. RNA sequencing was performed on skin and peripheral blood mononuclear cells (PBMCs). Twenty cytokines were quantified in blood plasma (n = 12).

**Results:**

Cluster analysis of the affected skin identified two CRPS subgroups (SG). SG1 exhibited increased gene expression related to epidermal development, metabolic processes, and a greater abundance of keratinocytes. SG2 showed enhanced transcriptomic changes in inflammatory, immune, and fibrotic processes, along with higher abundance of fibroblasts, macrophages, and endothelial cells. PBMCs transcriptomics revealed the same SG1/SG2 clusters and highlighted a stronger inflammatory response in the blood of SG1, suggesting distinct tissue-specific immune responses for the subgroups. Interleukin-1 receptor antagonist (IL-1RA) levels were higher in the blood plasma of SG1 (FDR = 0.01), consistent with its encoding gene *IL1RN* expression in PBMCs (log2 FC = 1.10, *P* < 0.001) and affected skin (log2 FC = 0.88, *P* = 0.006). Subgroups did not differ in demographic or clinical parameters but correlations among clinical factors varied between them.

**Conclusions:**

This study identified two potential biological subgroups of CRPS type 1 in women through skin and blood transcriptomic profiling, advancing the understanding of this condition. This could facilitate the development of targeted treatments for CRPS type 1.

**Supplementary Information:**

The online version contains supplementary material available at 10.1186/s10020-025-01148-y.

## Background

Complex regional pain syndrome (CRPS) is a primary pain condition that typically affects the limb, usually after an injury or trauma (Pons et al. 2015; Goebel 2011; Harden et al. 2010). It is characterized by intense and prolonged pain that is out of proportion to the severity of the initial injury. CRPS is more prevalent in females than males (4:1 ratio). Clinical manifestations are broad, encompassing sensory changes (allodynia, hyperalgesia), disturbed blood flow and sudomotor activity, motor dysfunction, and trophic changes in the affected limb (Harden et al. 2010; Maihöfner et al. 2007; Huge et al. 2011). Despite increasing research in the field, the underlying mechanisms remain elusive, and no definitive curative treatment exists (Zhu et al. 2024; Her et al. 2024; Ghaly et al. 2023).

The skin of CRPS-affected limbs shows changes in cell population, neuropeptide release, cytokine modulation, and immune responses, highlighting a role for the skin in CRPS pathophysiology (Kingery 2010; Birklein et al. 2018, 2014; Andronic et al. 2022; Mehling et al. 2024). For instance, rat fracture models of CRPS suggest that substance P and Calcitonin Gene-Related Peptide (CGRP) signaling activate receptors on the surface of keratinocytes, resulting in keratinocyte proliferation, and upregulation of inflammatory mediators such as Interleukin-1 and -6 (IL-1 and IL-6), Nerve Growth Factor (NGF), and Tumor Necrosis Factor-alpha (TNF-α) that lead to pain sensitization (Guo et al. 2012; Li et al. 2010; Wei et al. 2009; Sabsovich et al. 2008). Moreover, elevated levels of TNF-α, Macrophage Inflammatory Protein-1 beta (MIP-1β/CCL4), IL-2, IL-6, alongside reduced levels of IL-4, IL-8, and IL1-RA, in both skin and blood compared to control groups, suggest the involvement of systemic factors in CRPS (Lenz et al. 2013; Üçeyler et al. 2007). However, the precise contribution of these pro- and anti-inflammatory factors to the onset and maintenance of the condition remains poorly understood.

To date, various clinical classification models for CRPS have been proposed based on specific criteria. CRPS is traditionally divided into CRPS Type 1 (without confirmed nerve injury) and CRPS Type 2 (with confirmed nerve injury). Within CRPS Type 1, patients are often grouped into ‘cold’ or ‘warm’ phenotypes based on temperature changes in the affected limb, although these phenotypes may overlap with disease stages (e.g., the ‘cold’ phenotype is frequently associated with chronic phases) (Bruehl et al. 2016; Knudsen et al. 2023; Mangnus et al. 2023). Despite these frameworks, patient heterogeneity and overlaps between classifications highlight the complexity of CRPS. For instance, symptoms experienced by CRPS type I patients include sweating that might either increase or decrease, hair and nail growth can be either accelerated or slowed, and skin may become thinner or thickened (Harden et al. 2010; Borchers and Gershwin 2017). This variability extends to treatment responses across patients (Her et al. 2024), underscoring the need for more refined and comprehensive classification systems.

One approach is to incorporate molecular profiles into the classification system. Previously, an analysis of pro- and anti-inflammatory serum cytokines and their receptors clustered CRPS patients into distinct groups, suggesting the existence of biological subgroups based on molecular criteria (Alexander et al. 2012). Yet, it remains unclear whether these subgroups reflect changes happening locally within the affected tissues or are primarily driven by systemic inflammatory responses.

This study aimed to investigate whether biological subgroups of CRPS type 1 exist along the blood-skin axis that could explain the large heterogeneity. To this end, molecular profiles of CRPS female patients were examined by deep sequencing of skin and blood samples, as well as plasma cytokine quantification.

Our findings reveal the existence of two potential biological subgroups within CRPS Type I. This discovery lays the foundation for future studies where these results can be confirmed and further explored in larger cohorts. The identification of such subgroups could pave the way for the development of targeted treatment strategies, ultimately improving the management of this complex and challenging condition.

## Methods

### Study population

**Fig. 1:**
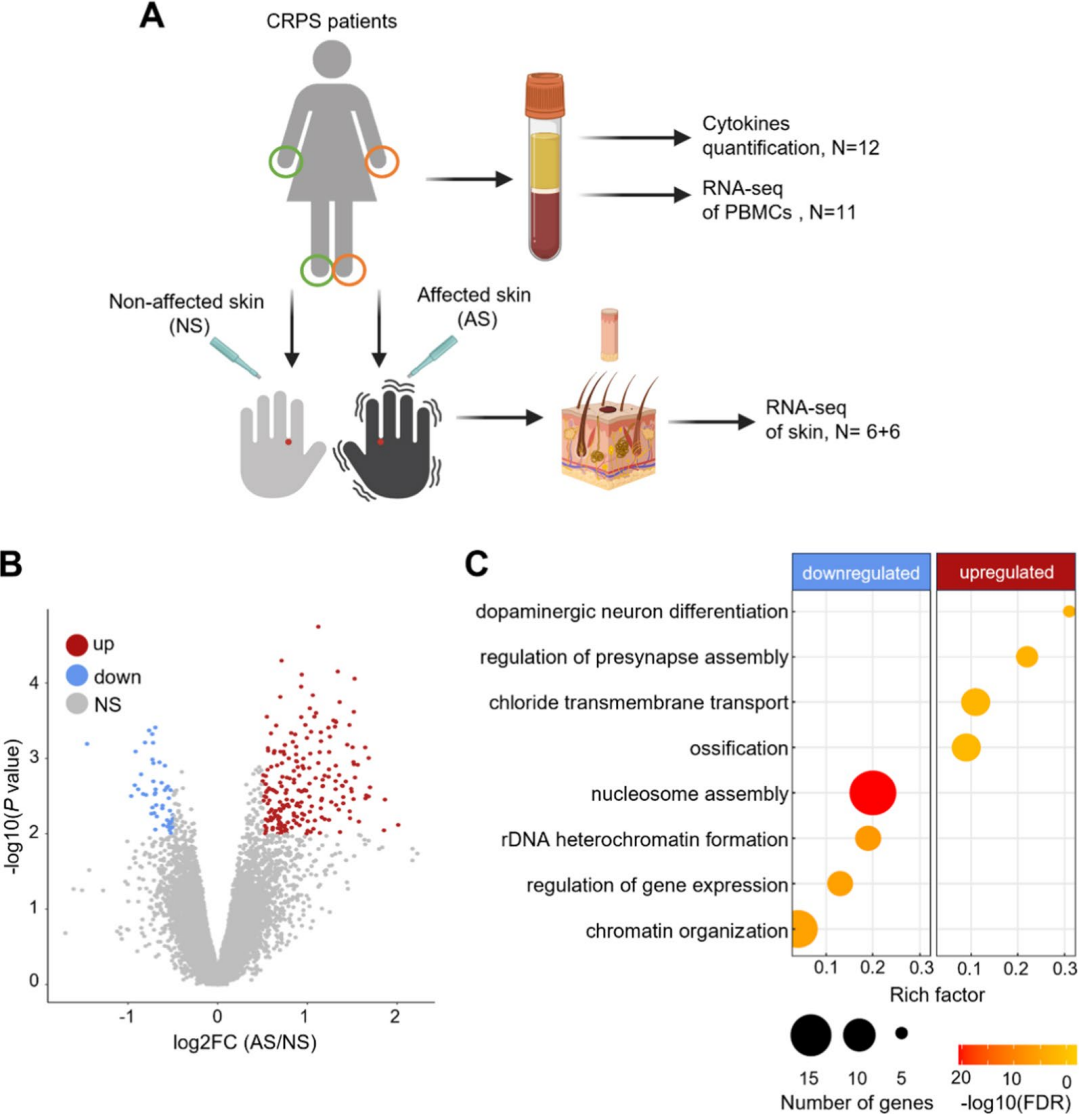
Transcriptomic profiles of skin biopsies from female CRPS patients. **A** Experimental Design. Twelve patients with unilateral CRPS affecting either the hand or foot donated peripheral blood for quantifying plasma cytokines and conducting bulk RNA sequencing (RNA-seq) of peripheral blood mononuclear cells (PBMCs). The first six patients also donated 3 mm skin punch biopsies from the CRPS-affected skin (AS) and contralateral non-affected skin (NS) for transcriptomic analysis. **B** Volcano plot displaying DEGs between AS over NS. Upregulated (up), downregulated (down), and non-significant (NS) DEGs are shown. **C** The top biological processes enriched by overrepresentation analysis in upregulated and downregulated DEGs from AS compared to NS (cutoff criteria of FDR < 0.05). The rich factor represents the ratio of DEGs associated with a pathway to the total number of genes annotated within that pathway

Given the known heterogeneity of CRPS, only female participants were included to reduce gender-related variability and enhance the consistency of the results. First, six women diagnosed with unilateral Complex Regional Pain Syndrome (CRPS) type 1, affecting either the hand or foot, were recruited for a skin biopsy. Participants were included regardless of whether the condition affected the right or left limb. In detail, Dr. Florian Brunner collected three-millimeter skin punch biopsies from the second interdigital space, carefully aiming to extract samples of consistent depth. Visual inspection did not show differences in sample thickness. Biopsies were collected from both, the affected and contra-lateral non-affected limbs at the exact same position for transcriptomic analysis (Fig. 1A). The six participants also donated six milliliters (mL) of peripheral blood in EDTA tubes. Due to its less invasive nature compared to skin biopsy, an additional six patients were included for blood collection. PBMCs from eleven participants were used for transcriptomic analysis, while blood plasma from all twelve participants was used for cytokine analysis. All participants met the Budapest criteria for CRPS diagnosis. Exclusion criteria were incomplete diagnosis or lack of consent, diagnosis of cancer, current infectious diseases (e.g. HIV, hepatitis), or being under guardianship. All study participants were over 18 years old. Participants completed demographics and pain questionnaires (Additional file 1: Table S1), which were recorded in RedCap^®^ (Harris et al. 2019; Harris et al. 2009). Clinical parameters between groups were compared with t-tests. For type of limb (hand, foot) Fisher Exact tests were calculated. Significance level was α = 0.05.

### Human skin and blood handling

Skin biopsies were transferred immediately after extraction into a 15 mL Falcon tube containing Dulbecco’s modified Eagle medium (DMEM) (Gibco, cat. no. 21331046) with 10% FCS and placed on ice for transportation. Subsequently, each biopsy was processed by immersion in 2 mL of RNAlater (Invitrogen, cat. no. AM7021) and stored in the refrigerator at 20 °C overnight, and then preserved at − 80 °C until further processing. Blood was centrifuged for 10 min at 700 rcf at 4 °C. The resulting plasma was aliquoted into 1.5 mL tubes, with each aliquot containing 300 microliters (μL), and stored at − 80 °C. The residual blood was diluted in 2 mL of PBS, carefully layered onto 3 mL of Ficoll-Plaque^™^ Plus (Cytiva, cat. no. 17144002), and centrifuged at 400 rcf at room temperature for 30 min. Subsequent steps involved the extraction of the PBMCs buffy coat, washing with PBS, centrifugation at 400 rcf at room temperature for 5 min, and resuspension of the resulting pellet in 700 μL of QIAzol Lysis Reagent (QIAGEN, cat. no. 79306). The final PBMCs product was stored at − 80 °C and later used for RNA sequencing (RNA-seq).

### Bulk RNA sequencing

Total RNA was extracted from skin biopsies and PBMCs with the RNeasy micro Kit (QIAGEN, cat. no. 217084) following the manufacturer's protocol, including on-column DNase treatment (QIAGEN, cat. no. 79254). RNA integrity number for all samples was assessed with an Agilent 2100 Bioanalyzer (Agilent Technologies) and is reported in Additional file 2: Table S1. Sequencing libraries were prepared according to the manufacturer’s instructions for the TruSeq Stranded mRNA library prep kit (Illumina) using 300 ng input of total RNA per sample. Size selection of libraries utilized AMPure XP Beads (Beckman Coulter), with quality verification on an Agilent 2100 Bioanalyzer. High-throughput sequencing of the RNAseq libraries for both skin and blood samples was performed on a Novaseq 6000 (Illumina), generating 20 M 100 bp single-paired reads per sample. FASTQ files were processed and analyzed on the SUSHI platform (Hatakeyama et al. 2016) of the Functional Genomic Center Zürich (FGCZ) of the University of Zurich and ETH Zurich. Read quality was assessed using FastQC, adaptor sequences at the 3’ end removed, and 4 bases at each end trimmed with fastp (v0.20). Filtered and trimmed sequences longer than 30 nt were pseudoaligned to the reference human genome build GRCh38.p13 (gene model definitions based on GENCODE release 37) using Kallisto (v0.46.1). Differentially expressed genes (DEGs) were determined using EdgeR (v3.22.1) generalized linear model. DEGs were defined as *P* < 0.01 and log2 fold change (log2 FC) ± 0.5. Principal Component Analyses were performed on normalized counts to identify sample stratification visually. Clustering was then verified using the Silhouette method, K-means, and hierarchical clustering. Cell types were approximated in the skin with deconvolution analysis using RNAseq databases with known cell frequencies (PRESS cohort (Skaug et al. 2019). The cell signature score plotted in the heatmaps ranged from -1 to 1. To calculate the percentage (%) of each cell signature, the mean score of each cell type per CRPS subgroup was determined. Then, a value of 1 was added to all scores to ensure they were only positive. To explore the enrichment of predefined gene sets or pathways within our DEG list, overrepresentation analysis was conducted using Bioconductor (v4.8.2) in R (v4.3) (R Core Team. R 2021). Biological terms were considered significant if the false-discovery rate (FDR) ≤ 0.01. Gene Set Enrichment Analysis (GSEA) was done on GSEA (v4.3.2) with default parameters. Hallmark gene sets were obtained from MSigDB and were significant if FDR ≤ 0.01.

### Cytokine quantification

Twenty plasma cytokines (G-CSF, GM-CSF, IFN-α2a, IFN-β, IFN-γ, IL-1β, IL-1RA, IL-4, IL-5, IL-6, IL-7, IL-8, IL-9, IL-10, IL-12p70, IP-10, MCP-1, MIP-1α, TNF-α, VEGF-A) were measured using chemiluminescence-based U-PLEX Viral Panel 1 Human Kit assays from Meso Scale Discovery (MSD, cat. no. K15343K-1) on a 1300 MESO^®^ QuickPlex SQ 120MM instrument (MSD) and DISCOVERY WORKBENCH^®^ 4.0 software. All plasma samples were analyzed in duplicates according to manufacturer protocols. Analyte concentrations were calculated from a standard curve using a four-parameter logistic fit using GraphPad Prism (v9.4.1) software. For graphing and analysis, concentrations below the detection limit were assigned the lower detection threshold, while concentrations exceeding the highest calibration standard were assigned the upper detection threshold. Normal distribution was tested with Shapiro–Wilk tests. Cytokine levels between two subgroups of CRPS were compared with t-tests in the case of normal distribution and with Mann–Whitney tests in the case of non-normal distribution. Unadjusted *P* values and FDR were calculated. Significance was defined as FDR < 0.05.

Blood plasma IL-1RA concentrations of CRPS subgroups were compared to previously measured blood plasma IL-1RA concentrations from an age- and sex-matched pain-free cohort of healthy volunteers (Additional file 1: Table S2) (Sirucek et al. 2024). IL-1RA concentrations were compared between CRPS subgroups and a CTRL group using ANOVA followed by Tukey Post-hoc test. *P* values were corrected for multiple comparisons with Bonferroni correction.

## Results

### Patient demographics

Twelve female patients with unilateral CRPS type 1 of the right hand (n = 4), left hand (n = 4), right foot (n = 3), and left foot (n = 1) were included in the study. Patient characteristics are summarized in Table 1. The mean age was 49.0 ± 13.8 years. Most patients underwent physical (physiotherapy, ergotherapy, or both) or medical (opiates, NSAR, metamizole, or topical) therapy (Additional file 1: Table S1). Eleven participants reported experiencing differences in temperature, discomfort, or mobility difficulties between their affected and non-affected limbs, and three participants reported having considered amputation of the affected limb at some point since the onset of their symptoms (Additional file 1: Table S1).

**Table 1:** Patient characteristics

Parameters	Value
Female	12 (100%)
Age, mean ± SD (in years)	49.1 ± 13.8
Patients with unilateral CRPS (%)	12 (100%)
Affected limb	
Hand (%)	8 (66.6%)
Right (%)	4 (50%)
Left (%)	4 (50%)
Foot (%)	4 (33.3%)
Right (%)	3 (75%)
Left (%)	1 (25%)
Duration of the symptoms since triggering event (in weeks), mean ± SD	74.9 ± 105.2
Skin temperature difference in °C, mean ± SD	− 0.1 ± 1.8
Current pain, mean ± SD (0–100)	32.0 ± 21.3
Maximum pain in the last 24 h, mean ± SD (0–100)	55.4 ± 21.7
Minimum pain in the last 24 h, mean ± SD (0–100)	25 ± 16.6
SFMPQ2, mean ± SD (0–600)	255 ± 104
BBPDS, mean ± SD (0–57)	16.2 ± 11.2
PCS, mean ± SD (0–52)	14.7 ± 11.3
CRPS severity score, mean ± SD (0–15)	10.3 ± 3.2

SD: Standard Deviation; SMFPQ2: Short-Form McGill Pain Questionnaire-2; BBPDS: Bath Body Perception Disturbance Scale; PCS: Pain Catastrophizing Scale

### Transcriptomic differences between affected and non-affected CRPS limbs

To profile the transcriptomic signatures of CRPS in the skin, we performed RNA-seq on biopsies from CRPS-affected skin (AS) and contralateral non-affected skin (NS) as a control (Fig. 1A). In total, 262 DEGs were found, with CRPS-affected limbs showing five times more upregulated genes than downregulated ones (Fig. 1B and Additional file 2: Table S2). Overrepresentation analysis revealed an upregulation of genes associated with dopaminergic neuron differentiation, regulation of presynapse assembly, chloride transmembrane transport, and ossification in AS compared to NS. Terms related to chromatin organization were downregulated (Fig. 1C and Additional file 2: Table S3). GSEA identified hallmark gene sets involved in cytokine signaling pathways, such as TNF signaling via NFκB, Interferon-γ response, and IL-6 pathway via activation of Janus Kinase 2 (JAK2)-signal transducer and Activator of Transcription 3 (STAT3) (IL-6/JAK/STAT3) to be enriched in AS compared to NS. Moreover, androgen response, epithelial-mesenchymal transition, and angiogenesis were overrepresented in AS (Table 2).

**Table 2:** GSEA of hallmark gene sets in comparative RNA-seq analysis of the skin

All affected over non-affected skin			Affected skin in SG2 over SG1			SG1: affected over non-affected skin			SG2: affected over non-affected skin		
GS	NES	FDR	GS	NES	FDR	GS	NES	FDR	GS	NES	FDR
TNF-α signaling via NFKB	2.34	< 0.001	Epithelial-mesenchymal transition	2.74	< 0.001	Androgen response	1.68	0.017	Epithelial-mesenchymal transition	2.91	< 0.001
KRAS signaling up	1.77	0.006	Angiogenesis	2.32	< 0.001	Oxidative phosphorylation	1.59	0.033	Interferon-γ response	2.48	< 0.001
Apical surface	1.77	0.004	Interferon-α response	2.22	< 0.001	Estrogen response early	1.58	0.024	Interferon-α response	2.43	< 0.001
Androgen response	1.76	0.003	Interferon-γ response	2.09	< 0.001	Epithelial-mesenchymal transition	− 2.22	< 0.001	TNF-α signaling via NFKB	2.31	< 0.001
Epithelial-mesenchymal transition	1.76	0.003	UV response down	2.07	< 0.001	E2F targets	− 1.81	0.004	Angiogenesis	2.31	< 0.001
Interferon-γ response	1.75	0.002	Coagulation	2.05	< 0.001				Coagulation	2.14	< 0.001
Angiogenesis	1.66	0.005	Apical junction	1.92	< 0.001				KRAS signaling up	2.11	< 0.001
IL-6/JAK/STAT3 signaling	1.64	0.005	KRAS signaling up	1.89	< 0.001				UV response down	2.01	< 0.001
E2F targets	− 2.68	< 0.001	IL-2/STAT5 signaling	1.86	< 0.001				Complement	1.98	< 0.001
G2M checkpoint	− 2.25	< 0.001	Complement	1.85	< 0.001				Inflammatory response	1.95	< 0.001
MYC targets v1	− 2.07	< 0.001	Inflammatory response	1.85	< 0.001				Allograft rejection	1.88	< 0.001
MYC targets v2	− 1.79	< 0.001	TNF-α signaling via NFKB	1.84	< 0.001				IL-6/JAK/STAT3 signaling	1.85	< 0.001
			Allograft rejection	1.83	< 0.001				IL-2/STAT5 signaling	1.83	< 0.001
			IL-6/JAK/STAT3 signaling	1.80	< 0.001				Apical junction	1.80	< 0.001
			Myogenesis	1.77	< 0.001				Myogenesis	1.74	< 0.001
			Apoptosis	1.65	0.002				Apical surface	1.72	< 0.001
			Hedgehog signaling	1.62	0.002				Hypoxia	1.64	0.003
			Oxidative phosphorylation	− 2.76	< 0.001				TGF-β signaling	1.57	0.005
			MYC targets v1	− 2.58	< 0.001				Oxidative phosphorylation	− 2.30	< 0.001
			MCY targets v2	− 1.92	< 0.001				E2F targets	− 2.21	< 0.001
			mTORC1 signaling	− 1.79	0.002				MYC targets v1	− 2.19	< 0.001
									G2M checkpoint	− 2.01	< 0.001
									MYC targets v2	− 1.78	0.002

GS: Gene Set; NES: Normalized Enrichment Score; FDR: False Discovery Rate

### Clustering analysis of affected skin transcriptome reveals two CRPS subgroups

**Fig. 2:**
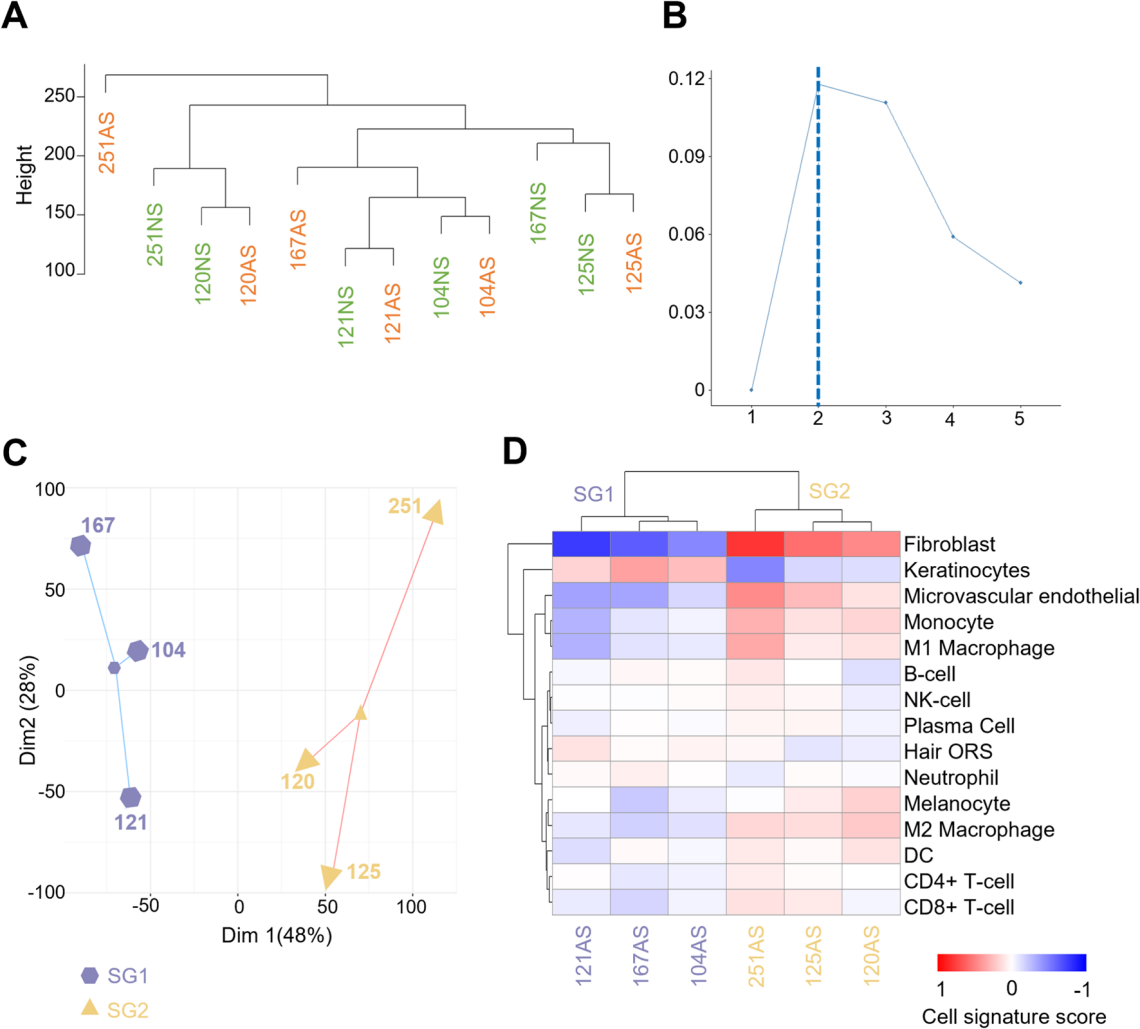
CRPS-affected skin clusters into two subgroups. **A** Hierarchical clustering dendrogram generated from RNA-seq in CRPS-affected skin (AS) and non-affected skin (NS). **B** Number of optimal clusters as proposed by Silhouette method. **C** K-means plot of AS normalized counts. **D** Deconvolution analysis of AS displaying patient stratification according to the cell signature score. NK-cell: Natural Killer cell; Hair ORS: Hair Outer Root Sheath; DC: dendritic cells

The transcriptome of skin samples did not reveal distinct clustering between AS and NS (Fig. 2A), prompting us to investigate potential subgroups within AS alone. Subsequent clustering analysis of AS identified the presence of two subgroups (SG), SG1 (n = 3) and SG2 (n = 3) (Figs. 2B–C). Non-affected skin did not cluster into the two subgroups (Additional file 3: Figs. S1A,B).

To assess differences in cell-type proportions between subgroups, we conducted a deconvolution analysis. Among the top five most abundant cell types, keratinocytes had a higher signature score in SG1 (SG1 = 0.27; 9.21% of all cells, SG2 = − 0.25; 4.57%) whereas SG2 had more fibroblasts (SG1 = − 0.62; 2.7%, SG2 = 0.61; 9.80%), microvascular endothelial cells (SG1 = − 0.28; 5.21%, SG2 = 0.28; 7.81%), monocytes (SG1 = − 0.14; 6.22%, SG2 = 0.20; 7.31%), and M1 macrophages (SG1 = − 0.30; 6.09%, SG2 = 0.18; 7.20%) (Fig. 2D and Additional file 4: Table S1), suggesting heightened fibrotic and immune response in SG2 compared to SG1. The consistent enrichment of these cell types in each subgroup was not observed in the non-affected skin (Additional file 3: Fig. S1C and Additional file 4: Table S1).

To identify subgroup-specific biological processes, five different comparative analyses were performed:

a AS-specific differences between subgroups

**Fig. 3:**
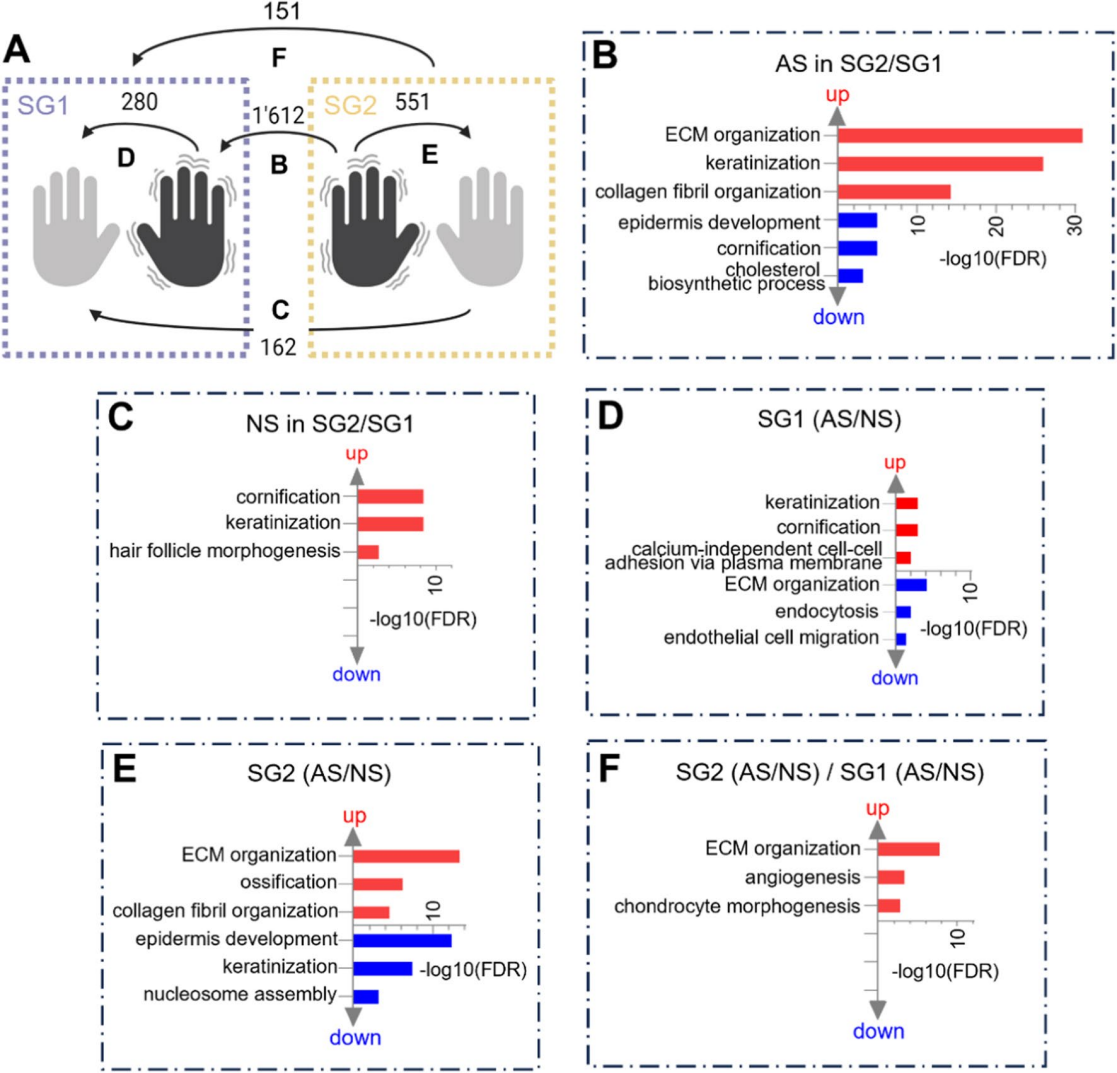
Comparison of DEGs and enriched biological processes across SG1, SG2, AS, and NS. **A** Diagram illustrating the number of DEGs obtained from comparative analyses between SG1 (purple), SG2 (yellow), AS (black limb), and NS (grey limb). **B**–**F** The top biological processes enriched in (**B**) SG2 (AS) over SG1 (AS), (**C**) SG2 (NS) over SG1 (NS), (**D**) SG1 (AS) over SG1 (NS), (**E**) SG2 (AS) over SG2 (NS), (**F**) SG2 (AS/NS) over SG1 (AS/NS). ECM: extracellular matrix

Comparing only the affected skin of SG2 to SG1 revealed 1612 DEGs (Fig. 3A and Additional file 2: Table S2). The top overrepresented processes in SG2 were extracellular matrix organization, keratinization, and collagen fibril organization. The top underrepresented terms were epidermis development, cornification, and cholesterol biosynthetic processes (Additional file 2: Table S3). GSEA identified enrichment of hallmark gene sets related to pro-fibrotic (epithelial-mesenchymal transition) and immune/inflammatory responses (Interferon-α and -γ response, IL-6/JAK/STAT3 signaling, IL2/STAT5 signaling, TNF signaling via NFκB, and complement) (Table 2). These results align with SG2 being characterized by a higher abundance of fibroblasts and immune cellular types from the deconvolution analysis (Fig. 2D). Downregulation of metabolic/proliferation gene sets, such as oxidative phosphorylation, MYC targets, and mechanistic Target of Rapamycin Complex 1 (mTORC1) signaling was found in SG2 compared to SG1 affected limbs (Table 2).

b NS-specific differences between subgroups

When comparing non-affected skin in SG2 to SG1, 162 DEGs were found (Fig. 3A and Additional file 2: Table S2). The analysis showed an overrepresentation of terms associated with keratinization (Fig. 3C, Additional file 2: Table S3), suggesting that patients in SG2 exhibited increased gene regulation related to keratinization processes in both AS (Fig. 3B) and NS biopsies (Fig. 3C) compared to SG1. Other enriched terms were cornification and hair follicle morphogenesis (Fig. 3C, Additional file 2: Table S3). No terms were underrepresented. No hallmark gene sets were significantly enriched by GSEA.

c Changes in affected vs. non-affected skin in SG1

When comparing AS to NS in SG1, 280 DEGs were identified (Fig. 3A and Additional file 2: Table S2). The top overrepresented biological processes were keratinization, cornification and calcium-independent cell–cell adhesion via plasma membrane (Fig. 3D, Additional file 2: Table S3). GSEA revealed that hallmark pathways related to androgen response, estrogen response, and oxidative phosphorylation were enriched (Table 2). Underrepresented terms were extracellular matrix organization, endocytosis, endothelial cell migration, and epithelial-mesenchymal transition (Fig. 3D and Table 2), indicating that the enhanced epidermal features observed in SG1 affected limb relative to SG2 are also evident when compared to its non-affected counterpart.

d Changes in affected vs. non-affected skin in SG2

The analysis revealed 551 DEGs, twice the number found in SG1 (Fig. 3A and Additional file 2: Table S2) suggesting more pronounced changes in SG2 between affected and non-affected limbs. Contrary to changes in SG1, extracellular matrix organization, and collagen fibril organization were the top enriched biological processes, while keratinization and epidermis development were underrepresented (Fig. 3E and Additional file 2: Table S3). GSEA highlighted the enrichment of pro-fibrotic changes (epithelial-mesenchymal transition) and inflammatory response (Interferon-α and -γ response, IL-6/JAK/STAT3 signaling, IL2/STAT5 signaling, TNF signaling via NFκB, and complement) in AS relative to NS. Downregulation of metabolic/proliferation gene sets (oxidative phosphorylation and MYC targets) was found (Table 2). Similar to the changes observed in SG2-affected limb relative to SG1-affected limb, heightened fibrotic and inflammatory features were found when SG2-affected limbs were contrasted with their non-affected counterparts.

e Changes in affected vs. non-affected skin between subgroups

When comparing SG2 to SG1 in the AS relative to NS, a total of 151 DEGs were found (Fig. 3A and Additional file 2: Table S2). Extracellular matrix organization, angiogenesis, and chondrocyte morphogenesis were enriched (Fig. 3F, Additional file 2: Table S3). No terms were underrepresented. No hallmark gene sets were significantly enriched by GSEA.

In summary, specific and distinct molecular processes were observed for each subgroup, both when comparing the affected and non-affected limbs, and when comparing only the affected limbs between subgroups. These results highlight the distinct nature of the subgroups while accounting for individual variability.

### PBMCs transcriptome also clusters into SG1 and SG2

**Fig. 4:**
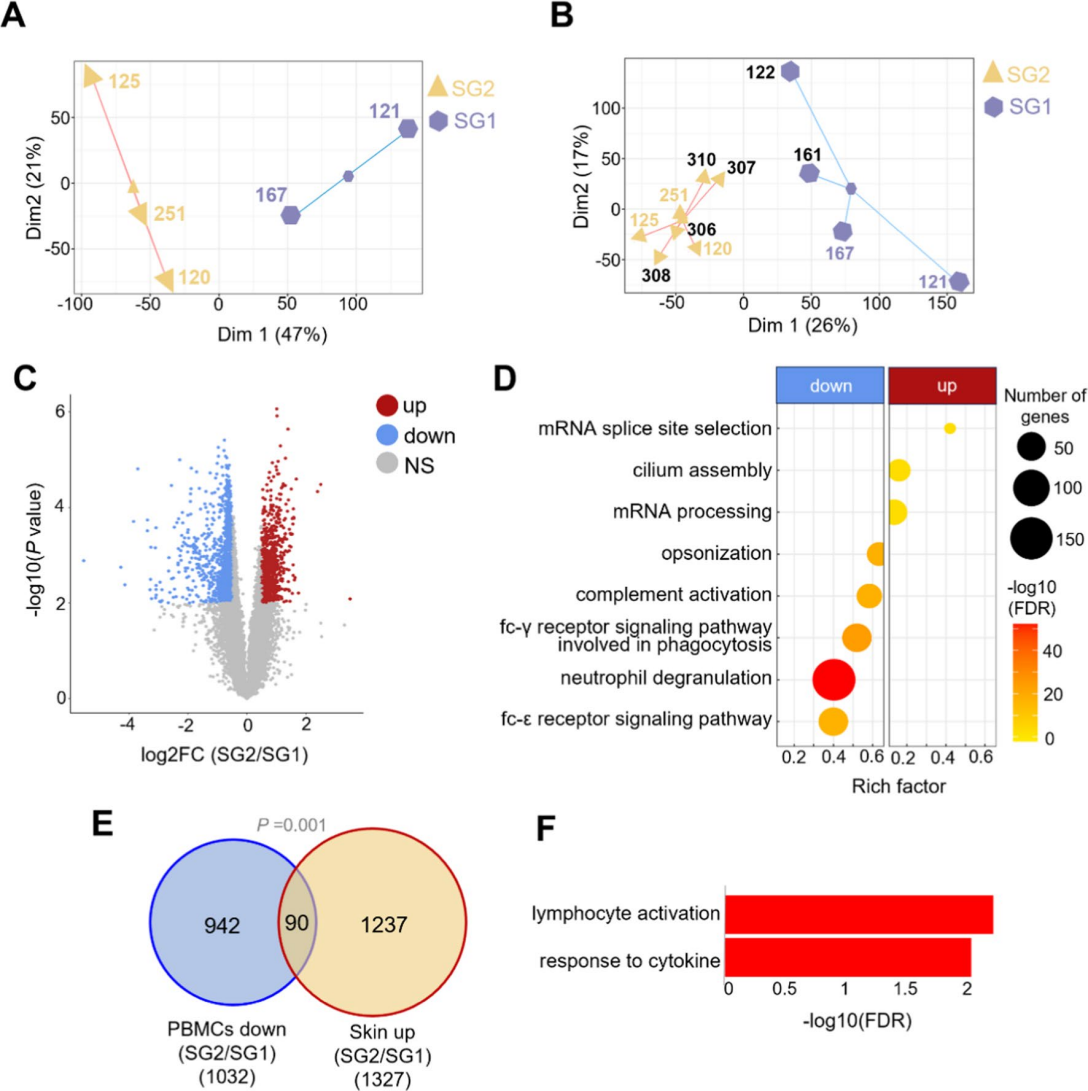
PBMCs transcriptome analysis supports the existence of SG1 and SG2 subgroups. **A** K-means clustering of normalized transcriptomic data from PBMCs of five CRPS donors whose skin was also analyzed. **B** K-means clustering of normalized transcriptomic data from 11 PBMCs CRPS samples. The ID numbers of the additional six patients added to the study are shown in black, while the ID numbers of the five patients with skin analysis are represented in yellow (SG2) and purple (SG1). **C** Volcano plot displaying DEGs from SG2 over SG1 PBMCs. Upregulated (up), downregulated (down), and non-significant (NS) DEGs are shown. **D** The top biological processes enriched by overrepresentation analysis in downregulated (down) and upregulated (up) DEGs from SG2 compared to SG1 PBMCs (cutoff criteria of FDR < 0.05). The rich factor represents the ratio of DEGs associated with a pathway to the total number of genes annotated within that pathway. **E** Overlap of downregulated DEGs (SG2/SG1) in PBMCs and upregulated DEGs (SG2/SG1) in the skin. *P* value indicates a statistical significance of the overlap (hypergeometric test). **F** The top biological processes enriched by overrepresentation analysis from the overlapped 90 genes of Fig. 4E

After analyzing the RNA-seq from skin samples, we further examined the PBMCs transcriptome from the same cohort of patients, excluding one individual from whom, for technical reasons, we could not isolate the PBMCs. Cluster analysis from PBMCs revealed the same patient distribution into SG1 and SG2 as observed in the skin transcriptome analysis (Fig. 4A). Adding PBMCs transcriptome data from six additional CRPS patients showed consistent clustering of patients into SG1 (n = 4) and SG2 (n = 7) (Fig. 4B). A total of 1887 DEGs were identified when comparing subgroups (Fig. 4C and Additional file 5: Table S1). Genes related to mRNA processing were enriched in SG2 (Fig. 4D and Additional file 5: Table S2). GSEA revealed underrepresentation of oxidative phosphorylation, mTORC1, MYC targets, E2F targets, and G2M checkpoint in the PBMCs of SG2 compared to SG1 (Table 3), consistent with skin analyses (Table 2).

**Table 3:** GSEA of hallmark gene sets in PBMCs RNA-seq (SG2/SG1)

GS	NES	FDR
Oxidative phosphorylation	− 3.02	< 0.001
Interferon-γ response	− 2.39	< 0.001
mTORC1 signaling	− 2.38	< 0.001
Interferon-α response	− 2.35	< 0.001
Reactive oxygen species pathway	− 2.32	< 0.001
Adipogenesis	− 2.31	< 0.001
Glycolysis	− 2.11	< 0.001
TNF-α signaling via NFKB	− 2.04	< 0.001
DNA repair	− 2.03	< 0.001
IL-6/JAK/STAT3 signaling	− 2.03	< 0.001
MYC targets v1	− 2.01	< 0.001
Cholesterol homeostasis	− 2.00	< 0.001
Fatty acid metabolism	− 1.98	< 0.001
Heme metabolism	− 1.95	< 0.001
E2F targets	− 1.95	< 0.001
Xenobiotic metabolism	− 1.94	< 0.001
Apoptosis	− 1.90	< 0.001
UV response up	− 1.88	< 0.001
Inflammatory response	− 1.85	< 0.001
Complement	− 1.83	< 0.001
Unfolded protein response	− 1.83	< 0.001
p53 pathway	− 1.79	0.001
Hypoxia	− 1.70	0.002
G2M checkpoint	− 1.59	0.005
Protein secretion	− 1.59	0.005
Estrogen response late	− 1.59	0.005
PI3K/AKT/mTOR signaling	− 1.58	0.005
Peroxisome	− 1.55	0.006

GS: Gene Set; NES: Normalized Enrichment Score; FDR: False Discovery Rate

However, opposite to the observations in skin transcriptomes, overrepresentation analysis and GSEA revealed a pronounced downregulation of immune response in PBMCs of SG2 compared to SG1, including neutrophil degranulation, Fc-ε and Fc-γ Receptor Signaling Pathway, TNF-α, and complement activation (Fig. 4D, Table 3 and Additional file 5: Table S2). Most DEGs between subgroups in the PBMCs transcriptome did not overlap with DEGs in the skin transcriptome (Additional file 3: Fig. S2A), suggesting distinct gene regulation patterns between the two tissues within subgroups. Nevertheless, we found a significant overlap of 90 genes (*P* = 0.001) that were downregulated in the PBMCs transcriptome but upregulated in the skin transcriptome of SG2 compared to SG1 (Fig. 4E and Additional file 6: Table S1). Overrepresentation analysis of these 90 genes showed enrichment of lymphocyte activation and response to cytokine processes (Fig. 4F, Additional file 6: Table S2). Any additional overlap between the two tissues across subgroups was not statistically significant (Additional file 3: Figs. S2B–D).

These findings suggest that the subgroups display distinct tissue-specific immune responses, with SG2 having a more pronounced immune response in the affected limb but showing downregulation of immune pathways in the blood compared to SG1.

### IL-1RA distinguishes CRPS subgroups across plasma, PBMCs, and skin

**Fig. 5:**
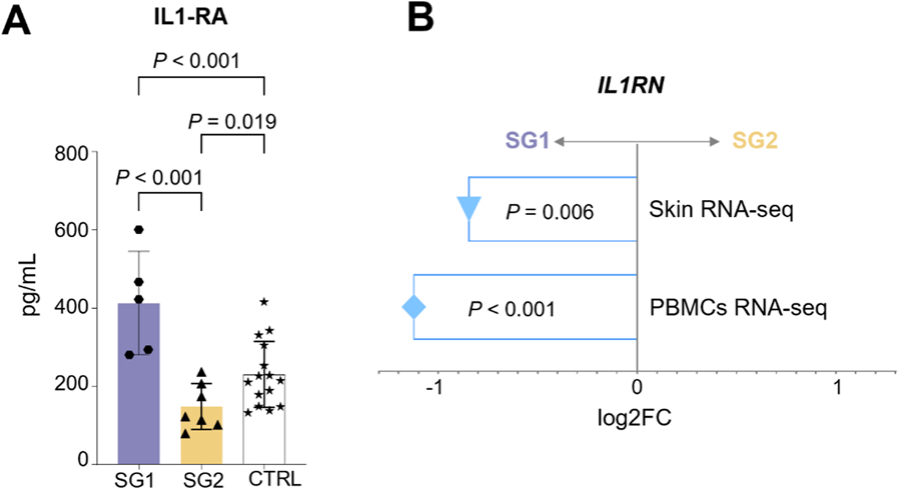
CRPS subgroups present different levels of IL-1RA and encoding gene (*IL1RN*) across tissues. **A** IL-1RA plasma levels in SG1 (n = 5), SG2 (n = 7), and healthy volunteers (CTRL, n = 15). P values are shown (ANOVA followed by Tukey Post-hoc test). **B** Differential expression of *IL1RN* between subgroups (SG) in skin and PBMCs RNA-seq. *P* values from the RNA-seq are shown

Having independently identified two CRPS subpopulations at a transcriptomic level in both skin and PBMCs, potential blood protein markers that could differentiate these populations were explored. A panel of twenty pro- and anti-inflammatory cytokines was measured in plasma samples from the twelve participants. Nine of twenty cytokines were detectable in the plasma of at least half of the patients (Table 4 and Additional file 7: Table S1). Plasma levels of IL-1RA, an inhibitor of the pro-inflammatory IL-1 family, were significantly lower in SG2 (n = 5) compared to SG1 (n = 7) (*P* < 0.001) (Fig. 5A). This pattern was confirmed at the transcriptional level by assessing the expression of the IL-1RA encoding gene*, IL1RN,* in the RNA-seq datasets. The results consistently showed downregulation of *IL1RN* in SG2 compared to SG1 in affected skin (log2 FC = − 0.85, *P* = 0.006) and PBMCs (log2 FC = − 1.15, *P* < 0.001) (Fig. 5B). Comparison of IL-1RA levels in the subgroups with plasma samples from 15 age-matched healthy female individuals (CTRL) showed that IL-1RA was significantly lower in SG2 than in the control group (*P* = 0.019), whereas its levels in SG1 were significantly higher (P < 0.001) (Fig. 5A).

**Table 4:** Cytokine levels in plasma samples from SG1 (n = 5) and SG2 (n = 7) patients

Cytokine	SG1: mean (ng) ± SD	SG2: mean (ng) ± SD	*P* value	FDR
IL-1RA	413.3 ± 118.0	148.7 ± 54.3	< 0.001	0.014
MCP-1	165.6 ± 72.5	87.8 ± 18.7	0.03	0.27
G-CSF	7.4 ± 6.2	2.6 ± 1.0	0.05	0.27
MIP-1α	16.1 ± 10.1	9.4 ± 2.6	0.14	0.44
IL-7	9.0 ± 6.9	4.1 ± 2.2	0.14	0.44
TNF-α	0.5 ± 0.3	0.3 ± 0.1	0.19	0.47
VEGF-α	111.0 ± 58.3	57.1 ± 49.7	0.27	0.54
IL-9	0.5 ± 0.5	0.3 ± 0.3	0.64	0.87
IP-10	286.3 ± 87.0	275.6 ± 131.9	0.89	0.94

*P* values and False Discovery Rate (FDR) are shown

### Clinical factors exhibit distinct correlations between subgroups

**Fig. 6:**
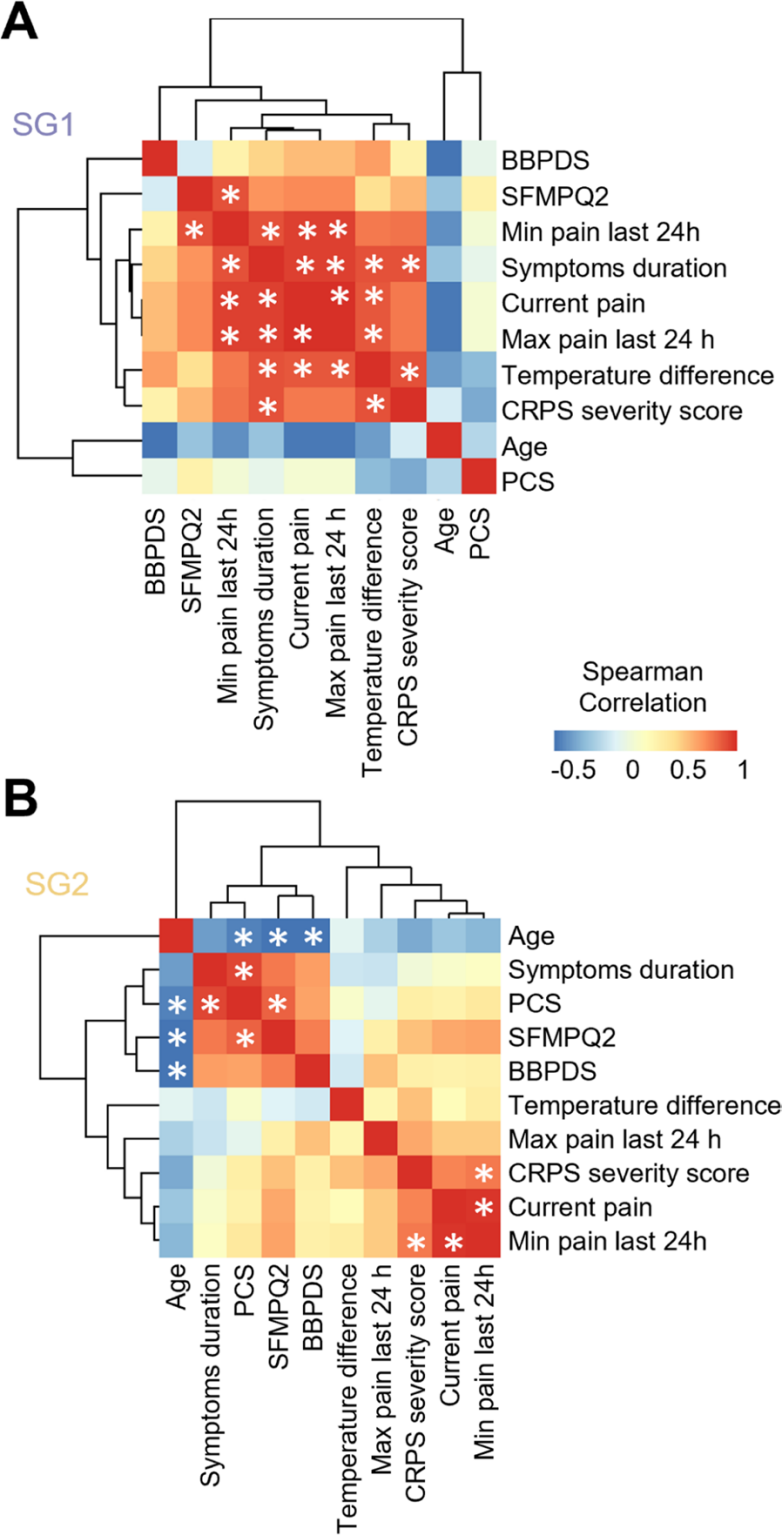
Correlation between demographic and clinical factors in each subgroup. Spearman Correlation in (**A**) SG1 and (**B**) SG2. The color gradient indicates Spearman’s ρ. *P < 0.05. SFMPQ2: Short-Form McGill Pain Questionnaire-2; BBPDS: Bath Body Perception Disturbance Scale; PCS: Pain Catastrophizing Scale; Temperature difference between affected and non-affected limbs

Considering the total of 12 patients analyzed in this study, no statistically significant differences were observed between SG1 and SG2 with respect to demographic factors or clinical parameters (Additional file 3: Fig. S3). To identify potential differences in clinical presentation between subgroups, correlations among these factors were calculated (Fig. 6 and Additional file 8: Table S1). SG1 patients showed a higher consistency in how they experience and perceive pain, as the levels of minimum pain within the last 24 h correlated positively with the current pain (ρ = 0.95, *P* = 0.014), maximum pain within the last 24 h (ρ = 0.95, *P* = 0.014), and the Short-Form McGill Pain Questionnaire-2 (SFMPQ2; ρ = 0.90, *P* = 0.037). Interestingly, the temperature difference of affected vs. non-affected limbs in SG1 correlated positively with levels of current pain (ρ = 0.88, *P* = 0.047), maximum pain within the last 24 h (ρ = 0.88, *P* = 0.047), CRPS severity score (ρ = 0.89, *P* = 0.041) and symptoms duration (ρ = 0.89, *P* = 0.041). In contrast, no significant correlation was found related to temperature difference in SG2. However, in SG2, symptoms duration correlated positively with Pain Catastrophizing Scale (ρ = 0.92, *P* = 0.003). Moreover, only in SG2 age correlated negatively with Pain Catastrophizing Scale (ρ = − 0.76, *P* = 0.049), SFMPQ2 (ρ = − 0.82, *P* = 0.023), and Body Perception Disturbance Scale (ρ = − 0.81, *P* = 0.027) scores (Fig. 6 and Additional file 8: Table S1).

## Discussion

Targeted treatment for CRPS is not available because the underlying pathomechanisms are poorly understood. This cross-sectional study is the first to describe the transcriptomic profile of affected and non-affected skin in patients with CRPS type 1. Transcriptomic analysis of skin identified two potential biological subgroups: SG1 and SG2. The analyses revealed that the subgroups differ in their cellular composition and several biological pathways related to immune, inflammatory, and metabolic responses. The subgroups were also distinguishable with PBMC transcriptomics and plasma IL-1RA levels.

### Common changes in the affected limbs across all CRPS patients

CRPS presents a significant challenge in both diagnosis and treatment, primarily due to its highly heterogeneous symptomatology. This data showed that when analyzing the transcriptomic profiles of six individuals with CRPS, the affected skin (AS) and non-affected skin (NS) did not cluster according to the condition, reinforcing the widely recognized heterogeneity of CRPS (Birklein et al. 2018; Knudsen et al. 2023; Mangnus et al. 2023; Alexander et al. 2012). Consequently, only a limited number of DEGs, 262 in total, were identified between AS and NS.

Common changes in all CRPS-affected skins were related to ossification, angiogenesis, and blood pressure, which could be related to bone turnover and vascular abnormalities identified in some CRPS patients (Kollmann et al. 2023; Wasner et al. 2001; Groeneweg et al. 2009; Huggenberger and Detmar 2011). Moreover, genes associated with IL-6 and TNF response, as well as chloride transmembrane transport, showed to be enriched in AS compared to NS, consistent with reported immune/inflammatory responses and sweating alterations in CRPS (Harden et al. 2010; Birklein et al. 2014; Üçeyler et al. 2007; Alexander et al. 2012). Dopaminergic neuron differentiation and presynapse assembly were also increased in AS. Previously, activation of dopaminergic neurons has been associated with responses to pain and individual variations in the pain experience (Scott et al. 2006).

It is important to note that SG1 and SG2 were not identified when exclusively clustering the contra-lateral non-affected skin samples. This discrepancy suggests two possible scenarios: 1) the disease induces distinct molecular alterations in the affected skin that are absent in the contralateral limb, or 2) individual variability in the systemic effects of the disease could influence the contralateral limb differently across patients. Although unilateral CRPS clinical assessment was guaranteed for us to be able to use the contralateral limb as a control, evidence for bilateral effects has been reported (Rasmussen et al. 2018; Maleki et al. 2000; Rijn et al. 2011), and we cannot exclude varying systemic impacts on NS limbs across individuals.

Although our initial goal was to investigate molecular differences within the patient population by comparing the AS to the NS, including comparisons with healthy controls in future studies would help clarify whether subgroup-specific changes represent deviations from normal physiological responses and address potential systemic effects comprehensively.

### Molecular signatures that characterize SG2 and SG1

The high variability among CRPS patients when analyzing the cohort without stratifying into subgroups caused many genes to fall below statistical significance, masking important genetic signatures. To address this, the affected skin transcriptomes were clustered, and two distinct subgroups were identified. SG1 showed a higher abundance of keratinocytes, increased cornification, and downregulation of pro-fibrotic processes, not only when compared to SG2 but also to their non-affected skin. Local keratinocyte proliferation and pathological thickening have been previously reported during the acute phase of CRPS (Birklein et al. 2014), but patients from SG1 were from both the acute and chronic phases. Indeed, two out of three patients showed longer symptom durations compared to SG2, suggesting that these might not be transient effects and likely represent features of an epidermal subtype.

SG2 showed a higher abundance of fibroblasts, macrophages, and endothelial cells in the affected skin, along with collagenous matrix reorganization, pro-fibrotic changes, and inflammation, indicating a dermal inflammatory-fibrotic subtype. Under acute inflammation, cells participating in the pro-inflammatory response typically undergo metabolic reprogramming to meet high energy demands, shifting from oxidative phosphorylation towards glycolysis to support cell proliferation and function (Heiden et al. 2009; Warburg 1979), a process observed in cancers but also in rheumatoid arthritis (Balogh et al. 2018). In SG2, a decrease in oxidative phosphorylation along with the downregulation of mTORC1 signaling, MYC, and E2F target genes in the affected skin was observed. These findings suggest a potentially impaired mitochondrial respiration and reduced proliferative capacity, raising the possibility that an energy switch to support cellular division might not be occurring. Therefore, the downregulation of oxidative phosphorylation could be a response to a more hypoxic cellular environment in inflamed tissue (Eltzschig and Carmeliet 2011; Akinsulie et al. 2023). This notion is supported by the fact that the biological process response to hypoxia was enriched in SG2 compared to SG1 in affected limbs (FDR = 0.018 [did not pass our stringent threshold of FDR less than 0.01], Rich factor = 0.16). Moreover, the term apoptosis was enriched in SG2 compared to SG1, suggesting a potential increase in cellular stress. Cell senescence, closely associated with impaired mitochondrial function (Lafargue et al. 2017; Yoon et al. 2006, 2003), as previously reported in CRPS (Tan et al. 2011; Higashimoto et al. 2008), could also contribute to the reduced oxidative phosphorylation observed in SG2. Further studies are needed to confirm the underlying mechanisms leading to a potential decreased cellular respiration and cellular division in SG2 skin cells.

Metabolic differences pointing at distinct mitochondrial functions between the two subgroups were also found in the transcriptomic data of PBMCs. Consistent with the skin, oxidative phosphorylation, mTORC1, and MYC targets were downregulated in SG2 compared to SG1.

Other biological processes related to inflammation and immune responses were enriched in the skin transcriptome of SG2 compared to SG1 but surprisingly, they were underrepresented in their PBMCs. Comparison of the gene expression profiles across subgroups and tissues revealed that most DEGs between SG2 and SG1 in the skin did not overlap with DEGs in the blood. This could be attributed to tissue-specific immune responses. Nevertheless, a set of 90 DEGs was identified as significantly overlapping between tissues, showing upregulation in the skin but downregulation in the blood of SG2 compared to SG1. These genes were associated with lymphocyte activation and cytokine response. Among these genes, Cluster of Differentiation 14 (CD14), a cell surface antigen known for the activation of innate immunity responses (Sharygin et al. 2023) was identified. CD14 is strongly expressed in monocytes and most tissue macrophages (McGovern et al. 2014), consistent with a higher abundance of monocytes and macrophages in the skin of SG2 relative to SG1. Possible explanations for the contrasting regulation of these genes between tissues might involve differential gene activation in response to skin damage between subgroups, and/or immune cell migration. A hypothesis for our observations is that in SG2, some immune cells such as monocytes, mobilize from the blood to the skin, where they subsequently exhibit enhanced expression of these genes. The lack of statistical significance among other comparisons such as DEGs being upregulated (or downregulated) in both tissues, supports this idea. Yet, the precise mechanisms that lead to contrasting patterns in CRPS subgroups between tissues remain unknown, and the biological relevance of these genes through functional analysis would require further studies.

The two subgroups did not differ in individual demographic or clinical parameters. Yet, correlations across clinical factors differed between subgroups suggesting that patients from the two subgroups may indeed have different clinical presentations when considering composite scores and testing larger cohorts. In SG1 patients, temperature differences in the affected limb relative to the non-affected might serve as an indicator of CRPS severity, duration, and pain intensity.

### Plasma IL-1RA can distinguish CRPS subgroups

IL-1RA and its encoding gene *IL1RN* were found to be consistently upregulated in plasma, PBMCs, and skin of SG1. IL-1RA inhibits the pro-inflammatory IL-1 signaling and is implicated in various inflammatory and autoimmune diseases (Horai et al. 2000; Colantuoni et al. 2023; Jacques et al. 2006). A higher concentration of IL-1RA in SG1 skin supports the less inflammatory skin phenotype compared to SG2. Furthermore, IL-1RA has been shown to promote keratinocyte proliferation (Kondo et al. 2013), which aligns with the higher abundance of keratinocytes in SG1 skin. Conversely, reduced levels of IL-1RA in SG2 agree with previous findings associating low IL-1RA expression with conditions promoting fibrosis and fibroblast activity (Park et al. 2018; Jarrell et al. 2022; Kanangat et al. 2006). Differences in IL-1RA between SG1 and SG2 echo a study that identified IL-1RA and IL-1β as significant discriminators in cytokine-based CRPS subgroups (Alexander et al. 2012**).** However, since IL-1β was not consistently detected in the patients from this study, a direct comparison between our subgroups and those in the previous study is not possible.

The assay of soluble biomarkers in peripheral blood represents the most feasible modality in clinical practice due to its ease of collection, reliability, and the availability of commercialized measurement kits. Our results suggest that IL-1RA is a cytokine capable of distinguishing subgroups of CRPS type 1, with a potential link to pathomechanistic skin changes. Notably, IL-1RA has been hypothesized as a biomarker for the radiographic severity of early knee osteoarthritis (Ma et al. 2020) and disability in multiple sclerosis (Blandford et al. 2021), highlighting its broader relevance across inflammatory and degenerative conditions. However, while our findings position IL-1RA as a potential blood marker to differentiate between subgroups, its validation as a reliable biomarker requires further investigation. Larger multi-center cohorts and comparative studies across different conditions are needed to confirm its diagnostic and prognostic utility. Larger cohorts might also reveal additional markers that can differentiate the subgroups, as a composite biomarker is likely necessary to accurately and robustly distinguish the subgroups from each other and from other diseases.

### Limitations

This study has limitations. Sample size: The sample size was small. Small sample size has the risk of leading to false positive results and might affect the lack of clinical distinctions between the identified groups. Therefore, it is important that these findings are replicated in larger cohorts before generalizing results. Gender-bias: Only female participants were included in this study to minimize gender-related variability. However, we cannot conclude whether similar subgroups exist in men or if sex differences affect the subgroups’ molecular distinctions. This should be explored in larger, independent cohorts. Lack of healthy controls for the skin scRNA-seq: This study aimed to investigate how the affected skin differs from the non-affected skin within the patient population itself. Nevertheless, we acknowledge that including healthy controls or other pain populations in future studies would provide valuable context to validate the subgroups and determine whether these transcriptomic patterns are specific to CRPS. Biopsy Depth: While we aim to control biopsy depth for consistency, variations in skin thickness could have influenced the observed differences in cell proportions between subtypes. However, consistent clustering of patients based on skin and blood profiles suggests that these subgroups reflect true biological differences rather than technical biases. Cross-sectional study: The cross-sectional study design limits our ability to make causal inferences, and further research is needed to determine whether the observed transcriptomic signatures reflect stable subtypes or dynamic states, that could be associated with disease flares or remission. Histology: Histological examination was not conducted that could corroborate the transcriptomic changes in tissue architecture. Despite the limitations mentioned, the consistency of the clusters between skin and blood samples, provides valuable insights into the molecular distinctions across CRPS patients, laying the groundwork for future studies that could address these limitations and deepen our understanding of the underlying mechanisms.

## Conclusions

**Fig. 7:**
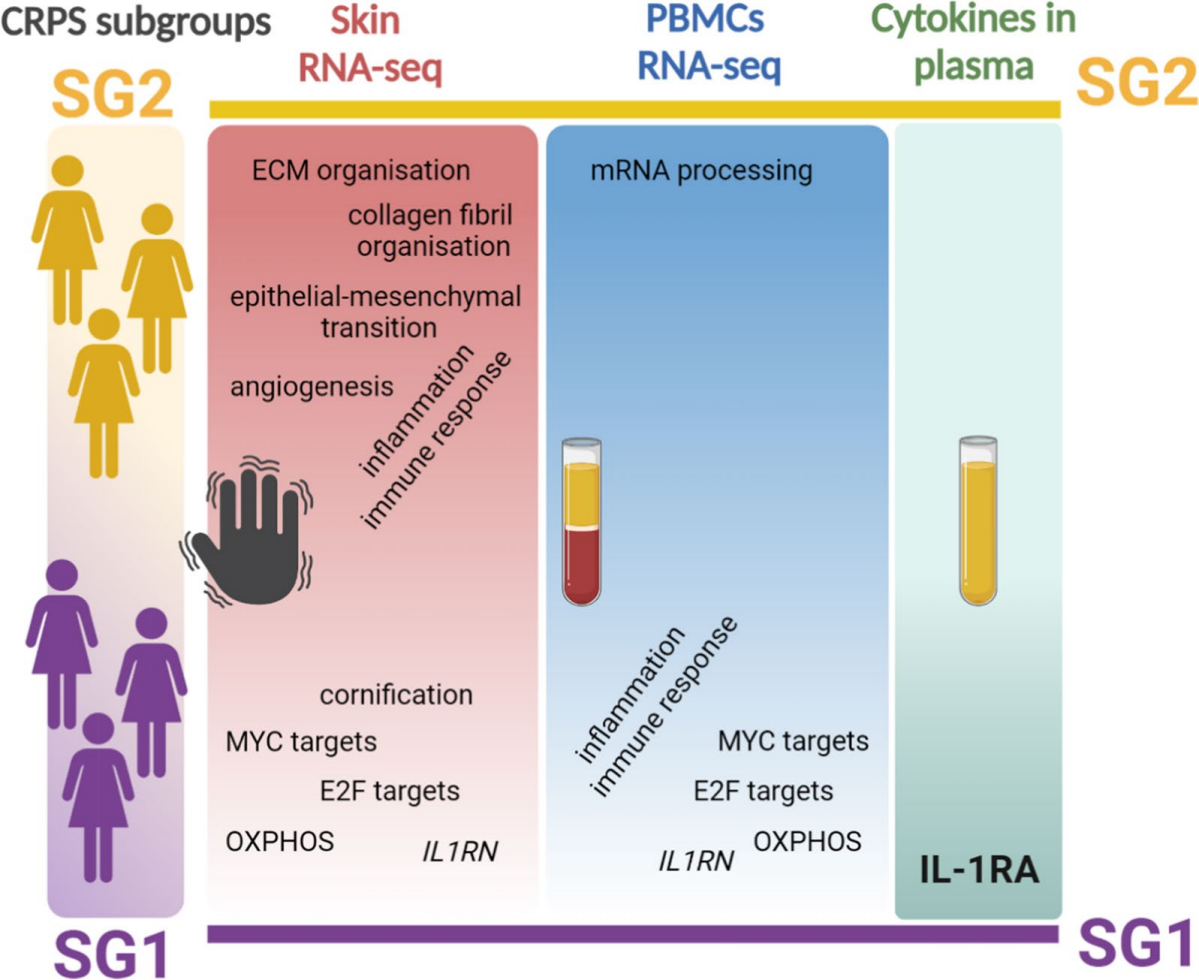
Summary of molecular signatures in CRPS subgroups 1 and 2 across tissues. The color gradient within each rectangle illustrates the relevance of the specified biological process for each subgroup. Darker red and blue colors denote that the process is overrepresented in SG2 skin and PBMCs, respectively, compared to SG1. A darker green color indicates higher IL-1RA plasma levels in SG1 relative to SG2. ECM: extracellular matrix; OXPHOS: oxidative phosphorylation; *IL1RN* and IL-1RA: Interleukin-1 Receptor Antagonist gene and protein; respectively. (Image created with BioRender.com)

This study identified two potential biological subgroups of CRPS type 1 in women along the skin-blood axis that exhibit contrasting molecular characteristics (Fig. 7). The opposing changes in the affected skin between subgroups could be relevant for developing more targeted treatment strategies for CRPS patients. The identification of IL-1RA as a potential blood marker may allow distinguishing between the two subgroups. While these findings may provide insight into the variability observed in previous CRPS studies, they require validation in larger, independent cohorts. Further research, including longitudinal studies, is necessary to fully understand the underlying mechanisms driving the existence and development of these subgroups, as well as to optimize treatment strategies tailored to each one of them.

## Supplementary Information

Additional file 1.

Additional file 2.

Additional file 3.

Additional file 4.

Additional file 5.

Additional file 6.

Additional file 7.

Additional file 8.

## Data Availability

The data for this study have been deposited in the European Nucleotide Archive (ENA) at EMBL-EBI under accession number PRJEB80812 (https://www.ebi.ac.uk/ena/browser/view/PRJEB80812). Any additional information required to re-analyze the data reported in this paper is available from the corresponding author upon request.

## Abbreviations

AS: Affected skin
BASEC: Business administration system for ethics committees
BBPDS: Bath body perception disturbance scale
CD14: Cluster of differentiation 14
CGRP: Calcitonin gene-related peptide
CRPS: Complex regional pain syndrome
DEGs: Differentially expressed genes
DMEM: Dulbecco’s modified eagle medium
ECM: Extracellular matrix
EDTA: Ethylenediaminetetraacetic acid
FDR: False-discovery rate
FGCZ: Functional genomic center zürich
Fig. S: Figure supplementary
HIV: Human immunodeficiency virus
G-CSF: Granulocyte colony-stimulating factor
GO: Gene ontology
GSEA: Gene set enrichment analysis
IL-1: Interleukin-1
IL-2: Interleukin-2
IL-6: Interleukin-6
IL-8: Interleukin-8
IL-1RA: Interleukin-1 receptor antagonist (protein)
IL1RN: Interleukin-1 receptor antagonist (gene)
IP-10/CXCL10: Interferon gamma-induced protein 10
JAK2: Janus kinase 2 signal transducer
MCP-1/CCL2: Monocyte chemoattractant protein-1
MIP-1β/CCL4: Macrophage inflammatory protein-1 beta
mTORC1: Target of rapamycin complex 1
NS: Non-affected skin
ORA: Overrepresentation analysis
PCA: Principal component analyses
PCS: Pain catastrophizing scale
PBMCs: Peripheral blood mononuclear cells
RNA-seq: RNA sequencing
SD: Standard deviation
SG1: Subgroup 1
SG2: Subgroup 2
STAT3: Activator of transcription 3
STAT5: Activator of transcription 5
SMFPQ2: Short-form mcgill pain questionnaire-2
Table S: Table supplementary
TNF-α: Tumor necrosis factor-alpha
VEGF-α: Vascular endothelial growth factor-alpha
