# Supplementary material for: Identification of two biological subgroups of complex regional pain syndrome type 1 by transcriptomic profiling of skin and blood in women
Source: Mol Med. 2025 Mar 12;31:94. doi: 10.1186/s10020-025-01148-y (PMC11900654; doi:10.1186/s10020-025-01148-y)
Supplement: Supplementary file 3 — Additional file 3. [file 10020_2025_1148_MOESM3_ESM.pdf]

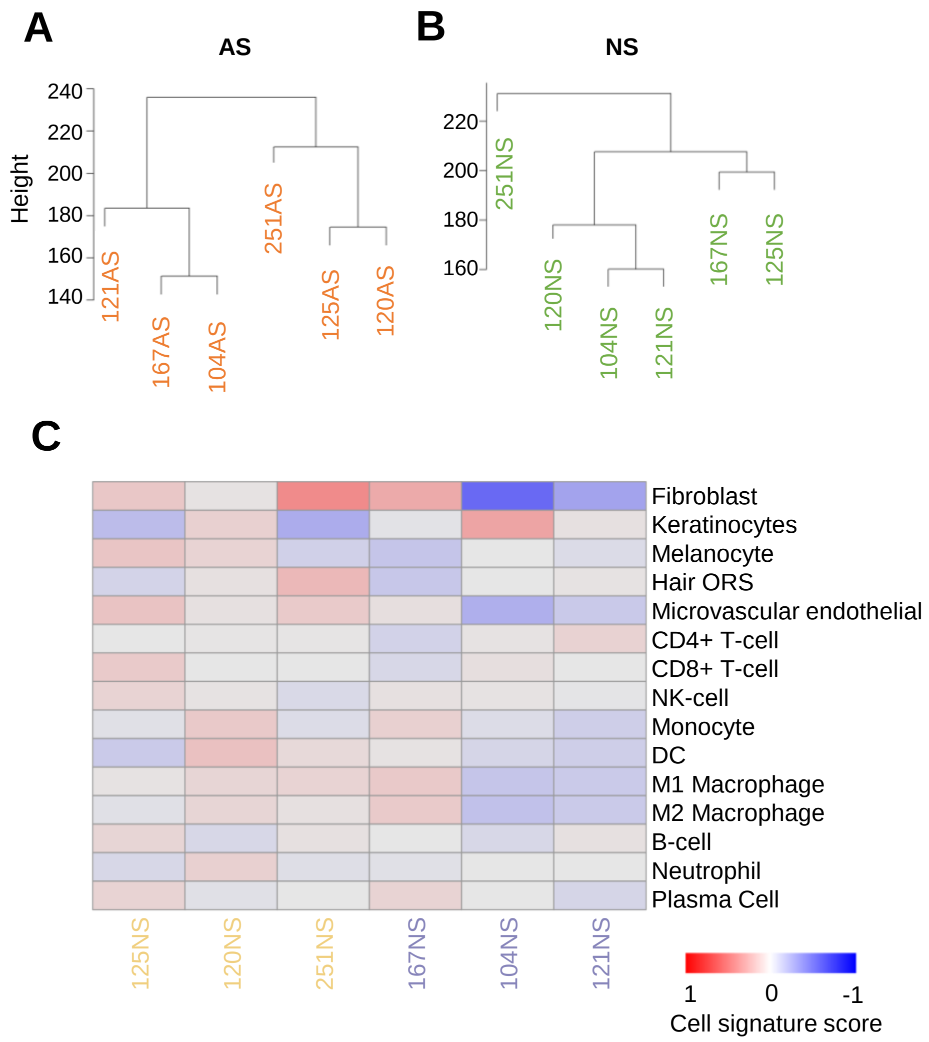

Figure S1. Distinct clustering of non-affected and affected skin samples. (A-B) Hierarchical clustering dendrogram generated from RNA-seq in (A) CRPS-affected skin (AS) and (B) non-affected skin (NS). (C) Deconvolution analysis of CRPS non-affected skin displaying patient stratification according to the cell signature score. Hair ORS= Hair Outer Root Sheath; NK-cell= Natural Killer cell; DC= dendritic cells.

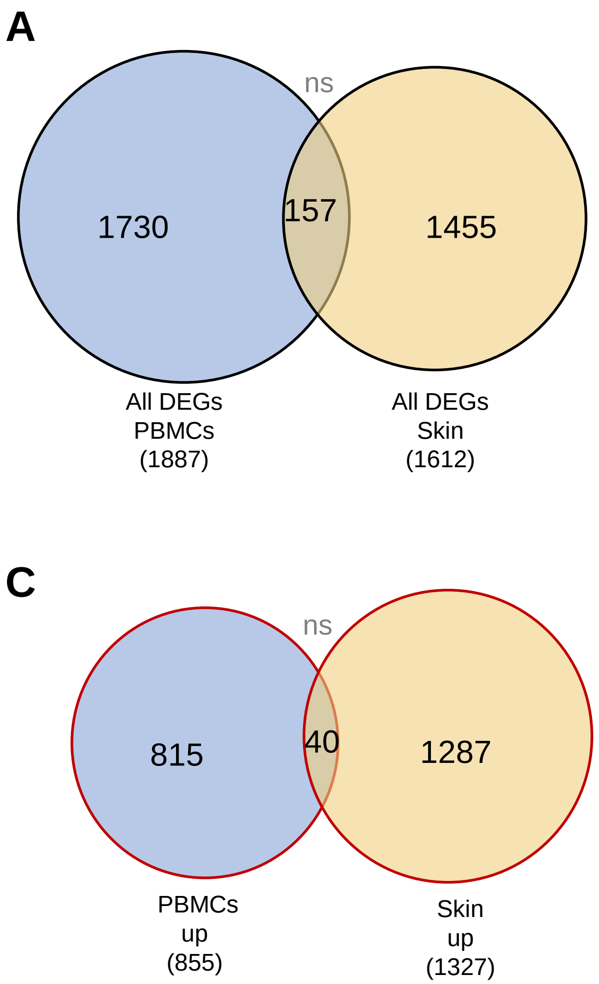

Figure S2. Comparison of DEGs between SG2 and SG1 in blood and skin. Overlap of (A) all DEGs (SG2/SG1) in blood and skin, (B) upregulated DEGs (SG2/SG1) in blood and downregulated DEGs (SG2/SG1) in skin, (C) upregulated DEGs (SG2/SG1) in both blood and skin, (E) downregulated DEGs (SG2/SG1) in both blood and skin. None of the comparisons showed statistical significance of the overlap (hypergeometric test). ns, not significant.

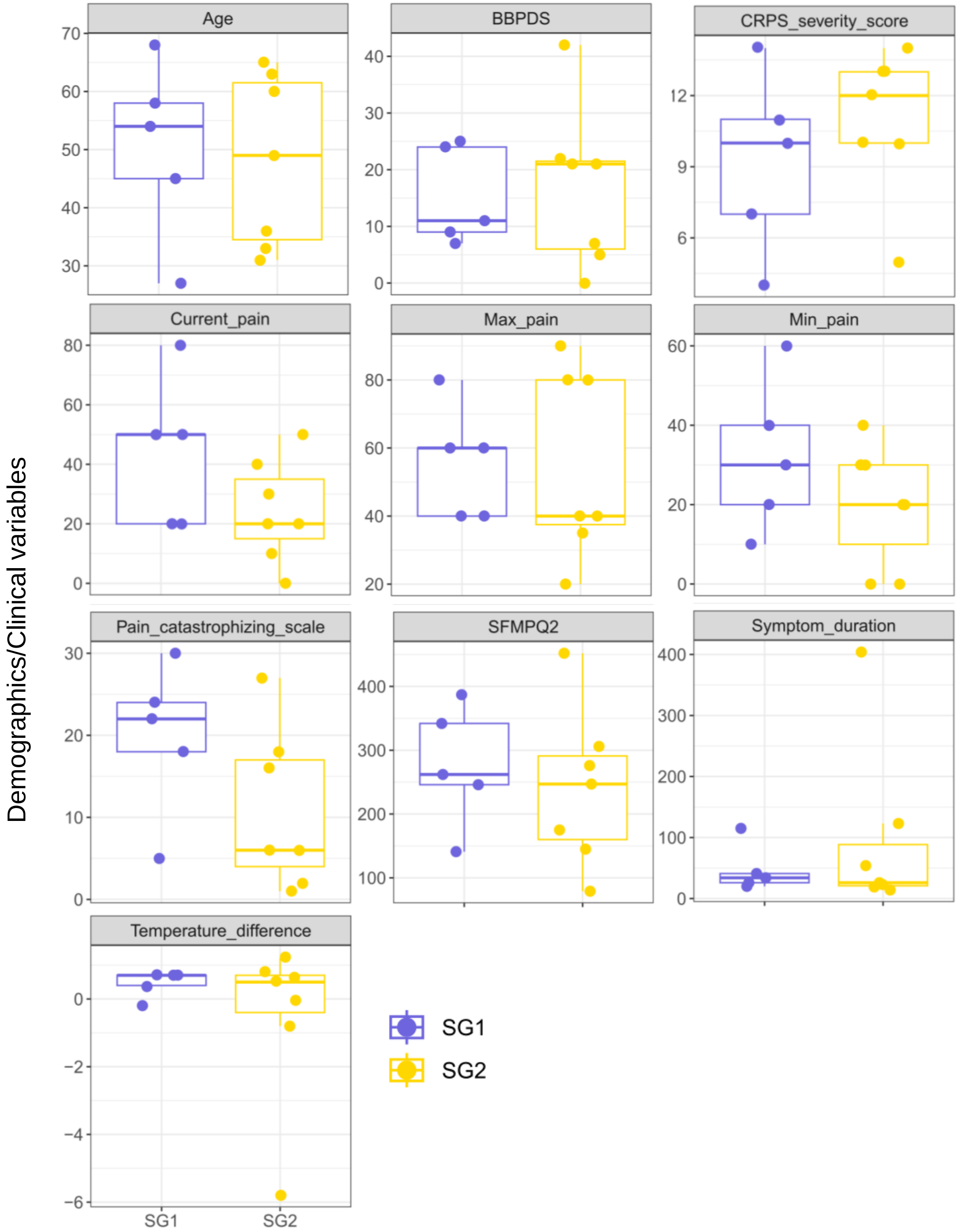

Figure S3. Comparison of demographics and clinical variables between subgroups. None of the variables showed statistical significance (t-test and Mann-Whitney U for normally and non-normally distributed variables, respectively). SFMPQ2=Short-Form McGill Pain Questionnaire-2; BBPDS= Body Perception Disturbance Scale; Temperature difference between affected and non-affected limb.
